# Nonlinear Robust Adaptive Multi-Modal Vibration Control of Bi-Electrode Micro-Switch with Constraints on the Input

**DOI:** 10.3390/mi8090263

**Published:** 2017-08-28

**Authors:** Mohsen Mohammadi, Mohammad Eghtesad, Hossein Mohammadi, Dan Necsulescu

**Affiliations:** 1 School of Mechanical Engineering, Shiraz University, Shiraz 71936, Iran; eghtesad@shirazu.ac.ir (M.E.); h_mohammadi@shirazu.ac.ir (H.M.); 2 Department of Mechanical Engineering, University of Ottawa, Ottawa, ON K1N 6N5, Canada

**Keywords:** multimodal design, active vibration control, unmodeled forces and impulse, robust adaptive

## Abstract

Micro functionally graded material (FGM) structures are able to have proper functions in vast environments. In this paper, nonlinear governing equations of the size-dependent micro-switch are derived using modified couple stress theory. Effective external forces including fringing field of electrostatic force and Casimir force are considered. Two electrodes cooperate to track the in-plane motions of the micro continuous system by tuning the supply voltages of the electrostatic force. An adaptive projection law is proposed to compensate for the effect of error in the initial estimates of system parameters. To achieve more reliability, a robust active vibration strategy is presented to withstand external disturbances. At any time, just one electrode is operational, and optimization is performed to decrease the controller gains. The highly nonlinear inputs have a singularity in the dynamics of the system, which are known as pull-in instability, so for safety, the controller gains are chosen such that the pull-in voltage is avoided. The dynamic response of the system is simulated using a single mode or multiple modes to validate the effectiveness of the presented vibration control approaches. The effects of error of the initial estimate of system parameters, the effect of impulse and the influences of various volume fractions are studied.

## 1. Introduction

Micro-Electro-Mechanical Systems (MEMS) have received extensive interest in the past two decades [1,2]. Due to their great advantages such as small size or mass, low cost of production, low power consumption, and easy integration into other systems, they are commonly used in numerous engineering devices, e.g., micro-actuators [3], micro-switches [4], atomic force microscopes [5], and micro-resonators [6]. Numerous analytical, numerical and experimental studies have been performed by researchers on the static and dynamic behaviors of such systems. Modeling of electrical and mechanical parts and their coupling is required in this field.

One of the important issues in the design of MEMS devices is the material selection with appropriate properties. Pure metals are of little use in such engineering applications because of the inconsistent properties. For instance, an application may require a material that is ductile as well as hard; there is no such material in nature. As a solution, the combination of one metal with other metals or non-metals (usually a ceramic) is used. Functionally Graded Material (FGM) can indeed be described as a class of advanced materials characterized by continuous variation of properties over volume. This type of material provides the specific benefits of both materials (metal and non-metal). The FGM concept originated in 1984 during the space plane project [7]. FGM structures play a significant role in various industrial fields, for instance energy engineering, optics, aerospace, nuclear energy, etc. [8]. Many kinds of research have already been reported on the static behavior [9], buckling analysis [10,11], contact problems [12], and free vibration behavior of FGM beams [13]. Most recently, Witvrouw and Mehta [14], Fu et al. [15], and Rahaeifard et al. [16] proposed the idea of using functionally graded materials in micro-electro-mechanical structures. Witvrouw and Mehta [14] designed and studied the process of producing MEMS structure layers made of polycrystalline silicon–germanium (poly-SiGe) on top of a standard 0.35 μm complementary metal-oxide-semiconductor (CMOS) process. Rahaeifard et al. [16] studied the resonant frequencies and sensitivities of first two modes atomic force microscope FGM cantilever.

Structures used in MEMS have a length on the order of microns. After many experimental studies, it seems that a new theory is needed to analyze such systems. The size-dependent static and vibration behaviors in micro-scales have experimentally been confirmed [17,18,19,20]. Torsion and tension experiments [21] and micro-bend test [22] have been designed to prove the size-dependent behaviors. The classical continuum mechanics theories cannot predict the size-dependent behaviors that occur in micro-scale structures. On the other hand, non-classical continuum theories such as the modified couple stress theory can interpret the size-dependent behaviors. Governing equations of motion for linear Euler–Bernoulli [23,24], nonlinear Euler–Bernoulli [25,26], linear Timoshenko [27], and nonlinear Timoshenko functionally graded micro-beams [25], using modified couple stress theory, have been derived. Farokhi et al. [28] presented a new size-dependent nonlinear model for the analysis of the behavior of carbon nanotube-based resonators. Ghayesh et al. [29] investigated the size-dependent dynamical performance of a microgyroscope via the use of the modified couple stress theory. Giunta et al. [30] proposed a unified formulation of one-dimensional beam models for the free vibration analysis of functionally graded (FG) beams. Via their approach, higher-order models that account for non-classical effects such as shear deformations and in- and out-of-plane warping can be formulated straightforwardly.

Many studies have been performed by different researchers on the vibration behavior of non-classical micro functionally graded beams. Mohammadi et al. [31] studied the mechanical behavior of an FG cantilever micro-beam subjected to nonlinear electrostatic pressure and temperature changes. Uncontrolled variation of electrostatic voltage may lead to instability in the dynamical behavior. Jia et al. [32,33] studied free vibration and the pull-in instability of functionally graded poly-SiGe micro-beams. Also, the free vibration characteristics of micro-switches under combined electrostatic, intermolecular forces and axial residual stress have been studied [34].

Proper performance of many MEMS devices depends on appropriate use of MEMS actuators. Magnetic, piezoelectric, thermal, optical and electrostatic devices are examples of on-chip actuators in MEMS devices. These actuators are used to achieve better dynamical behavior. There are many types of research concerning the control of MEMS [35,36,37,38,39,40]. The aims of the control algorithms are to establish a regulation [36,37,40] or a tracking [41] control law based on the linear [38,40] or the nonlinear [39,41,42] dynamic model of the micro-beam, where the supply voltage is considered a control input. MEMS are driven in both open-loop and closed-loop procedures. In addition, techniques for vibration absorbing can be divided into passive, semi-active and active control. The final step for improving accuracy and speed of response in the active vibration control is the introduction of feedback, e.g., closed-loop control. Sun et al. [43] designed a closed-loop controller for a 2-DOF capacitive force sensor. Sun et al. [44] studied the design, fabrication and control problem of a two-axis electrostatic micro-actuator. Nguyen and Krylov [41] developed a feedback controller to suppress the vibrations of a micro-electro-mechanical clamped-free beam operated at parametric resonances.

Active vibration control usually involves stability and robustness issues. In this paper, robust active vibration control of a functionally graded micro-switch is investigated. The boundary conditions of the micro-switch are assumed to be clamped-clamped (C-C) or clamped-free (C-F) and the system is under a combined action of Casimir force and electrostatic force in the framework of the modified couple stress theory. The supply voltage is considered the control input, and the vibration amplitude and the velocity are considered the outputs of the system. A robust adaptive feedback controller, which guarantees the proper transient dynamic response as well as steady state response while the system is under the influence of unknown bounded forces and the dimensionless parameters are unknown, is presented. This control algorithm ensures zero output tracking error when parameters are constant, and disturbances are zero. It also guarantees zero regulation error when the parameters are time-varying, and the dynamical disturbances are bounded. Several numerical simulation results are presented to verify the analytical results. Uncertainty in the system parameters is considered in these simulations. The performance of the designed control is shown for single-mode and multi-mode vibrational system.

## 2. Dynamic Modeling and Equations of Motion

### 2.1. Continuous Modeling

**Figure 1:**
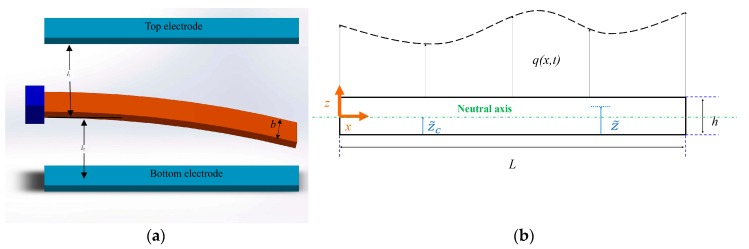
Functionally graded material (FGM) micro-switch model and the electrodes configuration: (**a**) clamped-free boundary condition and (**b**) diagram of the micro-switch.

As presented in Figure 1, the micro-switch is modeled as a functionally graded Euler–Bernoulli micro-beam with cross section area A, length L, width b and thickness h is considered. The variable z indicates the distance of a point from the neutral axis. It should be noted that the physical neutral plane is not the same as the geometric middle plane for the functionally graded micro-beams, due to the inhomogeneous material properties in the lateral direction [23]. It is assumed that the functionally graded beam properties $\Gamma \left(\tilde{z}\right)$ vary along the lateral coordinate based on a power law, i.e.,

(1) $$\Gamma \left(\tilde{z}\right)=\Gamma_{1}+{\left(\frac{\tilde{z}}{h}\right)}^{n}\left(\Gamma_{2}-\Gamma_{1}\right)$$

where Γ=ρ, μ, E and ν, represent the density, the shear modulus, the elastic modulus and the Poisson’s ratio, respectively; subscripts 1 and 2 refer to the two basic constituent materials, usually a ceramic and a metal. The power index n determines the type of the variation of the properties along the lateral direction.

Using the modified couple stress theory and the Hamilton principle, the following non-dimensional governing equation of motion is obtained [33]:

(2) $$\alpha_{1}\frac{\partial^{2}\bar{w}}{\partial {\bar{t}}^{2}}+\alpha_{2}\frac{\partial^{4}\bar{w}}{\partial {\bar{x}}^{4}}+\delta \alpha_{3}\frac{\partial^{2}\bar{w}}{\partial {\bar{x}}^{2}}\int_{0}^{1}{\left(\frac{\partial \bar{w}}{\partial \bar{x}}\right)}^{2}d\bar{x}+\alpha_{4}\frac{\partial \bar{w}}{\partial \bar{t}}+\delta \alpha_{5}\frac{\partial^{2}\bar{w}}{\partial {\bar{x}}^{2}}=\bar{q}$$

where δ=1 for the clamped-clamped (C-C) boundary conditions and δ=0 for the clamped-free (C-F) boundary conditions. Some authors [45,46,47] studied the effects of curvature-related and inertial-related nonlinearities on the vibrational response for cantilever beams. These effects are not considered in this paper. The normalized external lateral force per unit length, $\bar{q}$, is expressed as

(3) $$\bar{q}=\frac{A}{{\left[{\bar{l}}_{b}-\bar{w}\right]}^{2}}{\bar{V}}_{b}^{2}+\frac{B}{\left[{\bar{l}}_{b}-\bar{w}\right]}{\bar{V}}_{b}^{2}-\frac{A}{{\left[{\bar{l}}_{t}+\bar{w}\right]}^{2}}{\bar{V}}_{t}^{2}-\frac{B}{\left[{\bar{l}}_{t}+\bar{w}\right]}{\bar{V}}_{t}^{2}+\frac{C}{{\left[{\bar{l}}_{b}-\bar{w}\right]}^{4}}-\frac{C}{{\left[{\bar{l}}_{t}+\bar{w}\right]}^{4}}$$

In Equations (2) and (3) $\bar{x},\;\bar{t},\;\bar{w},\;{\bar{V}}_{t}$ and ${\bar{V}}_{b}$ are the non-dimensional form of longitudinal position, time, transverse deflection, the applied voltage for the top electrode and the bottom electrodes, respectively.$\;{\bar{l}}_{b}$ and ${\bar{l}}_{t}$ denote normalized distances between the FGM micro-switch from the top electrode and the bottom electrode, respectively. The electrostatic forces considering the first fringing field correction are $A{\bar{V}}_{t}^{2}/{\left[{\bar{l}}_{t}-\bar{w}\right]}^{2}+B{\bar{V}}_{t}^{2}/\left[{\bar{l}}_{t}-\bar{w}\right]$ and the Casimir force is denoted by $C/{\left[{\bar{l}}_{t}-\bar{w}\right]}^{4}$ [33].$\;A,\;B,\;C,\;\alpha_{1},\;\alpha_{2},\;\alpha_{3},\;\alpha_{4},\;\alpha_{5}$ are constants that depend on material properties and the geometry of the micro-switch. These variables are defined as follows:

(4) $$\bar{x}=\frac{x}{L},\;\bar{w}=\frac{w}{h},\;\bar{t}=\frac{t}{t_{c}}\;\alpha_{1}=\frac{mh}{t_{c}^{2}},\;\alpha_{2}=\frac{Hh}{L^{4}},\;\alpha_{3}=-\frac{H_{1}h^{3}}{2L^{4}},\;\alpha_{4}=\frac{c_{d}h}{t_{c}},\;\alpha_{5}=-\frac{N_{a}h}{L^{2}}\;m=\int_{A}\rho \left(\tilde{z}\right)dA,\;H_{1}={\left(EA\right)}_{eq}=\int_{A}\hat{E}\left(z\right)dA,\;H_{2}={\left(EQ\right)}_{eq}=\int_{A}\hat{E}\left(z\right)zdA\;H_{3}={\left(EI\right)}_{eq}=\int_{A}\hat{E}\left(z\right)z^{2}dA,\;H_{4}={\left(\mu A\right)}_{eq}=\int_{A}\hat{\mu }\left(z\right)dA\;H=H_{1}{\tilde{z}}_{c}^{2}-2H_{2}{\tilde{z}}_{c}+H_{3}+H_{4}l^{2}-\frac{{\left(H_{1}{\tilde{z}}_{c}-H_{2}\right)}^{2}}{H_{1}}\;\;A=\frac{\varepsilon_{0}b}{2h^{2}},\;B=\frac{0.65\varepsilon_{0}}{2h},\;C=\frac{\pi^{2}\bar{h}cb}{240h^{4}}$$

in which the effective modulus is obtained as $\hat{E}=E$ for a narrow beam (b<5h) and $\hat{E}=E/\left(1-\nu^{2}\right)$ for a wide beam (b≥5h). The variable $\bar{h}=1.055\times 10^{-34}\;\mathrm{Js}$ is the reduced Planck’s constant, $c=3\times 10^{8}\;{\mathrm{ms}}^{-1}$ is the speed of light in a vacuum, and $t_{c}$ is the time constant. The variables $t_{c},\;c_{d},\;N_{a}$ and l are the time constant, the damping coefficient, the axial residual force and the material length scale parameter, respectively.

The boundary conditions for the C-C micro-beam become

(5) $$\bar{w}\left(0\right)=\frac{\partial \bar{w}}{\partial \bar{x}}\left(0\right)=\bar{w}\left(1\right)=\frac{\partial \bar{w}}{\partial \bar{x}}\left(1\right)=0$$

and the boundary conditions for C-F are

(6) $$\bar{w}\left(0\right)=\frac{\partial \bar{w}}{\partial \bar{x}}\left(0\right)=\frac{\partial^{2}\bar{w}}{\partial {\bar{x}}^{2}}\left(1\right)=\frac{\partial^{3}\bar{w}}{\partial {\bar{x}}^{3}}\left(1\right)=0$$

The initial conditions are

(7) $$\bar{w}\left(\bar{x},0\right)=\frac{disp\left(x\right)}{h}=\bar{disp}\left(\bar{x}\right)\;{\frac{\partial \bar{w}}{\partial \bar{t}}|}_{t=0}=\frac{vel\left(x\right)}{L}t_{c}=\bar{vel}\left(\bar{x}\right)$$

In the following, the overbar notation will be omitted for brevity.

### 2.2. Discretized Modeling

The discretized form of the partial differential equation of motion (2) and boundary conditions (5) and (6) can be obtained using modal analysis technique. The transversal deflection of the switch can be acquired by a convergent series of infinite terms and can be approximated by a series of finite terms. The number of terms of the series specifies the approximation’s accuracy. The finite term series could be easily used in a simulation program. The deflection is approximated by sum of finite smooth functions as

(8) $$w\left(x,t\right)=\overset{m}{\underset{i=1}{\sum }}\xi_{i}\left(t\right)\phi_{i}\left(x\right)$$

where $\xi_{i}\left(t\right)$ is the time-varying generalized displacement of the ith mode. $\phi_{i}\left(\cdot \right)$ is the continuous mode shape function of the ith mode and these functions are orthogonal over the domain [0,1]. The ith vibrational mode shape function of C-C boundary conditions (Equation (5)) takes the form

(9) $$\phi_{i}\left(x\right)=E_{i}\left[\left(\cos\left(s_{i}\right)+\cosh\left(s_{i}\right)\right)\sin\left(s_{i}x\right)-\left(\sin\left(s_{i}\right)+\sinh\left(s_{i}\right)\right)\cos\left(s_{i}x\right)\;\;-\left(\cosh\left(s_{i}\right)+\cos\left(s_{i}\right)\right)\sinh\left(s_{i}x\right)+\left(\sinh\left(s_{i}\right)+\sin\left(s_{i}\right)\right)\cosh\left(s_{i}x\right)\right]$$

in which $s_{i}$ is a solution of equation $\cos\left(s_{i}\right)\cosh\left(s_{i}\right)=1$. The ith mode vibrational shape function for the boundary conditions (6) can be obtained as

(10) $$\phi_{i}\left(x\right)=E_{i}\left[\left(\cos\left(s_{i}\right)+\cosh\left(s_{i}\right)\right)\sin\left(s_{i}x\right)-\left(\sin\left(s_{i}\right)+\sinh\left(s_{i}\right)\right)\cos\left(s_{i}x\right)\;\;-\left(\cosh\left(s_{i}\right)+\cos\left(s_{i}\right)\right)\sinh\left(s_{i}x\right)+\left(\sinh\left(s_{i}\right)+\sin\left(s_{i}\right)\right)\cosh\left(s_{i}x\right)\right]$$

where $s_{i}$ is a solution of equation $\cos\left(s_{i}\right)\cosh\left(s_{i}\right)=-1$.

For both sets of boundary conditions, the coefficient $E_{i}$. in mode shapes are chosen such that

(11) $$\int_{0}^{1}{\left(\phi_{i}\left(x\right)\right)}^{2}dx=1$$

and the natural frequencies are related to $s_{i}$ as

(12) $$\omega_{i}=s_{i}^{2}\sqrt{\frac{H}{mL^{4}}}$$

The first five modes $s_{i}$ (i=1,…,5 ), for C-C and C-F boundary conditions are presented in Table 1.

To obtain the discretized form, first, both sides of Equation (2) are multiplied by the function $\phi_{i}\left(\cdot \right)$ and then an integrating is performed over the set [0,1]. The new governing equations of motion are derived in the matrix form of

(13) $$M\ddot{\xi }\left(t\right)+G\dot{\xi }\left(t\right)+K\xi \left(t\right)=U\left(\xi ,V_{b},V_{t}\right)$$

where $\xi \left(t\right)=\left[\xi_{1}\left(t\right),\ldots ,\xi_{m}\left(t\right)\right]$ is the time-varying generalized displacement vector. In this case, K and G are m×m matrixes. These matrices are obtained as follows

(14) $$M\left(i,j\right)=\delta_{ij}\alpha_{1}\int_{0}^{1}{\left({\hat{\phi }}_{i}\left(x\right)\right)}^{2}dx=\delta_{ij}\alpha_{1}$$

(15) $$G\left(i,j\right)=\delta_{ij}\alpha_{4}\int_{0}^{1}{\hat{\phi }}_{i}^{2}dx=\delta_{ij}\alpha_{4}$$

(16) $$K_{1}\left(i,j\right)=\delta_{ij}\alpha_{2}\int_{0}^{1}{\left({\hat{\phi }}_{i}^{\prime \prime }\left(x\right)\right)}^{2}dx$$

(17) $$K_{2}\left(i,j\right)=\delta_{ij}\delta \alpha_{3}f\left(\xi \right)\int_{0}^{1}{\left({\hat{\phi }}_{i}^{\prime }\left(x\right)\right)}^{2}dx$$

(18) $$K_{3}\left(i,j\right)=\delta_{ij}\delta \alpha_{5}\int_{0}^{1}{\left({\hat{\phi }}_{i}^{\prime }\left(x\right)\right)}^{2}dx$$

(19) $$K\left(i,j\right)=K_{1}\left(i,j\right)+K_{2}\left(i,j\right)+K_{3}\left(i,j\right)$$

where

(20) $$f\left(\xi \right)=\overset{m}{\underset{k=1}{\sum }}\xi_{k}^{2}\left(t\right)\int_{0}^{1}{\left({\hat{\phi }}_{k}\left(r\right)\right)}^{2}dr=\overset{m}{\underset{k=1}{\sum }}\xi_{k}^{2}\left(t\right)$$

The function in Equation (20) introduces nonlinearity in the governing equations of motion and the initial conditions for the Equation (13) are evaluated as

(21) $$\xi_{i}\left(0\right)=\int_{0}^{1}disp\left(x\right){\hat{\phi }}_{i}\left(x\right)dx\;{\dot{\xi }}_{i}\left(0\right)=\int_{0}^{1}vel\left(x\right){\hat{\phi }}_{i}\left(x\right)dx$$

where $\xi \left(t\right)={\left[\xi_{1}\left(t\right),\ldots ,\xi_{m}\left(t\right)\right]}^{T}$ and $\dot{\xi }\left(t\right)={\left[{\dot{\xi }}_{1}\left(t\right),\ldots ,{\dot{\xi }}_{m}\left(t\right)\right]}^{T}.$

By the use of the binomial series and the multi-nominal expansion, the nonlinear terms in Equation (3) due to Casimir and the electrostatic forces can be expanded as

(22) $$\frac{1}{{\left(g\pm w\right)}^{2}}=\frac{1}{2}\overset{\infty }{\underset{k=0}{\sum }}\underset{k_{1}+k_{2}+\ldots +k_{n}=k}{\sum }\frac{\left(k+1\right)!g^{-2-k}}{k_{1}!k_{2}!\ldots k_{m}!}{\left(\mp 1\right)}^{k}\overset{m}{\underset{i=1}{\prod }}{\left(\xi_{i}\phi_{i}\right)}^{k_{i}}$$

(23) $$\frac{1}{g\pm w}=\overset{\infty }{\underset{k=0}{\sum }}\underset{k_{1}+k_{2}+\ldots +k_{n}=k}{\sum }\frac{k!g^{-1-k}}{k_{1}!k_{2}!\ldots k_{m}!}{\left(\mp 1\right)}^{k}\overset{m}{\underset{i=1}{\prod }}{\left(\xi_{i}\phi_{i}\right)}^{k_{i}}$$

(24) $$\frac{1}{{\left(g\pm w\right)}^{4}}=\frac{1}{6}\overset{\infty }{\underset{k=0}{\sum }}\underset{k_{1}+k_{2}+\ldots +k_{n}=k}{\sum }\frac{\left(k+3\right)!g^{-4-k}}{k_{1}!k_{2}!\ldots k_{m}!}{\left(\mp 1\right)}^{k}\overset{m}{\underset{i=1}{\prod }}{\left(\xi_{i}\phi_{i}\right)}^{k_{i}}$$

To simplify the forcing function vector $U\left(\xi ,V_{b},V_{t}\right)$, three sets of functions are defined as

(25) $$F_{1,\pm }\left(k_{1},\ldots ,k_{m};i\right)=\frac{1}{2}\int_{0}^{1}\frac{\left(k+1\right)!g^{-2-k}}{k_{1}!k_{2}!\ldots k_{m}!}{\left(\mp 1\right)}^{k}\phi_{i}\overset{m}{\underset{j=1}{\prod }}\phi_{j}^{k_{j}}dx\;={\left(\mp 1\right)}^{k}\frac{1}{2}\frac{\left(k+1\right)!g_{0}^{-2-k}}{k_{1}!k_{2}!\ldots k_{m}!}\int_{0}^{1}\phi_{i}\left(x\right)\overset{m}{\underset{j=1}{\prod }}\phi_{j}^{k_{j}}\left(x\right)dx$$

(26) $$F_{2,\pm }\left(k_{1},\ldots ,k_{m};i\right)=\int_{0}^{1}\frac{k!g^{-1-k}}{k_{1}!k_{2}!\ldots k_{m}!}{\left(\mp 1\right)}^{k}\phi_{i}\overset{m}{\underset{j=1}{\prod }}\phi_{j}^{k_{j}}dx\;={\left(\mp 1\right)}^{k}\frac{k!g_{0}^{-1-k}}{k_{1}!k_{2}!\ldots k_{m}!}\int_{0}^{1}\phi_{i}\overset{m}{\underset{j=1}{\prod }}\phi_{j}^{k_{j}}dx$$

(27) $$F_{3,\pm }\left(k_{1},\ldots ,k_{m};i\right)=\frac{1}{6}\int_{0}^{1}\frac{\left(k+3\right)!g^{-4-k}}{k_{1}!k_{2}!\ldots k_{m}!}{\left(\mp 1\right)}^{k}\phi_{i}\overset{m}{\underset{j=1}{\prod }}\phi_{j}^{k_{j}}dx\;={\left(\mp 1\right)}^{k}\frac{1}{6}\frac{\left(k+3\right)!g_{0}^{-4-k}}{k_{1}!k_{2}!\ldots k_{m}!}\int_{0}^{1}\phi_{i}\overset{m}{\underset{j=1}{\prod }}\phi_{j}^{k_{j}}dx$$

The external forcing vector is

(28) $$\begin{array}{cc} U\left(i\right)=\overset{\infty }{\underset{k=0}{\sum }} & \underset{k_{1}+k_{2}+\ldots +k_{n}=k}{\sum }\xi_{1}^{k_{1}}\ldots \xi_{m}^{k_{m}}[A\;V_{b}^{2}F_{1,-}\left(k_{1},\ldots ,k_{m};i\right)+BV_{b}^{2}F_{2,-}\left(k_{1},\ldots ,k_{m};i\right)\\ & +CF_{3,-}(k_{1},\ldots ,k_{m};i)-AV_{t}^{2}F_{1,+}\left(k_{1},\ldots ,k_{m};i\right)\\ & -BV_{t}^{2}F_{2,+}\left(k_{1},\ldots ,k_{m};i\right)-CF_{3,+}\left(k_{1},\ldots ,k_{m};i\right)] \end{array}$$

The series in Equation (28) may only be expanded for four terms in this paper. As will be stated in the control algorithm, there is no need for state space presentation of Equation (13). For the numerical simulation procedure, Equation (13) is presented in state space form as

(29) $${\dot{x}}_{1}=x_{2}\;{\dot{x}}_{2}=-M^{-1}Kx_{1}-M^{-1}Gx_{2}+M^{-1}U\left(x_{1},V_{b},V_{t}\right)+M^{-1}D$$

where $x={\left[x_{1}\;x_{2}\right]}^{T}$ is the state vector of size 2 m.

## 3. Active Vibration Control of a Nonlinear System

Let us consider the nonlinear governing equation of motion (Equation (13)) with the desired smooth tracking signal $\xi_{d}\left(t\right)$, which is designed so that the deflection of the micro-switch follows a desired signal. The tracking signal is continuous, has a continuous derivative and is bounded in time. The material properties are assumed to be unknown and possibly time varying but belong to a closed set. The nominal values of properties and the sets’ bounds are expected to be known.

An adaptive robust control algorithm will be designed for the nonlinear FGM micro-beam. In this algorithm, vibrations of the micro-beam are being absorbed while the parameters of the system are unknown and unmodeled external forces affect the system performance. In the next section, the control algorithm for functionally graded material micro-switch’s dynamics model is simulated to verify its effectiveness.

The governing equations of motion, as discussed in the previous section, could be written as

(30) $$M\left(p_{M}\right)\ddot{\xi }\left(t\right)+G\left(p_{G}\right)\dot{\xi }\left(t\right)+K\left(p_{K}\right)\xi \left(t\right)=U\left(\xi ,V_{b},V_{t}\right)+D\left(t,\xi \right)$$

where D denotes the vector of disturbances that acts independently in every channel of the system’s inputs. In the active vibration control of the micro-beam system, D represents unknown external forces or an unmodeled dynamic. The vector of disturbance forces D is assumed to be bounded. The vector of unknown parameters in the governing equations of motion is denoted by p and is divided in three parts, $p={\left[p_{M}^{T}\;p_{G}^{T}\;p_{K}^{T}\right]}^{T}$, where $p_{M}$, $p_{G}$, and $p_{K}$ denote the unknown parameters in matrixes M, G, and K, respectively. The unknown parameter p enters linearly in the matrixes as in the governing equations of motion (see Equations (14)–(19)). The vector p is assumed to be bounded and belongs to a neighborhood with nominal value $p_{N}$ and radius $p_{R}$. In other words, $\;\|p_{M}-p_{MN}\|\leq p_{MR}$, $\|p_{G}-p_{GN}\|\leq p_{GR}$, and $\|p_{K}-p_{KN}\|\leq p_{KR}$, for some known values $p_{MN}$, $p_{MR}$, $p_{GN}$, $p_{GR}$, $p_{KN}$, and $p_{KR}$. In the case of active vibration control, $p_{M}=\alpha_{1}$, $p_{G}=\alpha_{4}$, and $p_{K}={\left[\alpha_{2}\;\alpha_{3}\;\alpha_{5}\right]}^{T}$.

It should be noted that M is constant (but unknown) and diagonal with positive arrays in these research’s cases, such that M is positive definite with known bounds.

To achieve zero tracking error, new state variables are introduced

(31) $${\tilde{x}}_{1}\left(t\right)=\xi \left(t\right)-\xi_{d}\left(t\right)$$

(32) $${\tilde{x}}_{2}={\dot{\tilde{x}}}_{1}+{\Lambda \;\tilde{x}}_{1}=\left(\dot{\xi }+\Lambda \;\xi \right)-\left({\dot{\xi }}_{d}+{\Lambda \;\dot{\xi }}_{d}\right)=\dot{\xi }-{\dot{\xi }}_{r}$$

in which ${\dot{\xi }}_{r}={\dot{\xi }}_{d}-\Lambda \;{\tilde{x}}_{1}$ and Λ is an arbitrary positive definite matrix. Note that if ${\tilde{x}}_{1}$ tends to zero, $\tilde{\xi }\left(t\right)$ will converge to $\xi_{d}\left(t\right)$.

To evaluate the control algorithm’s performance for a system with unknown parameters and bounded disturbance, two penalty signals are introduced in what follows. These signals optimize the energy usage and the vibration overshoot.

(33) $$ℋ=\left[\begin{array}{c} Q^{\frac{1}{2}}\;\tilde{x}\\ R^{\frac{1}{2}}\;U \end{array}\right]$$

in which $\tilde{x}$ is ${\left[{\dot{\tilde{x}}}_{1}^{T}\;{\tilde{x}}_{1}^{T}\right]}^{T}$. R>0 and Q≥0 are weighting matrixes.

The solution to the problem of robust adaptive control of the system is defined as follows.

**Definition** **1.** *The adaptive tracking control is said to be globally solvable for the equations of motion (30), if for any smooth and bounded tracking signal* $\xi_{d}\left(d\right)$ *with continuous time derivative, at least, up to order two, a state feedback law*

(34) $$U=U\left(\xi ,\dot{\xi },\xi_{d},{\dot{\xi }}_{d},\hat{\phi },\;t\right)$$

*exists such that*

*(i)*  $\left\|\hat{p}\right\|$, ‖ξ‖ *and* $\left\|\dot{\xi }\right\|$ *are bounded for any* t≥0.
*(ii)*  *for any bounded disturbance* D(ξ,t) *and* T≥0, *the following inequality holds for some positive numbers* γ, α, $\epsilon_{0}$ *and* V(0)
  (35) $$\int_{0}^{T}\|ℋ\left(t\right)\|dt\leq 2V\left(0\right)+\gamma^{2}\int_{0}^{T}\left(\begin{array}{c} \|D^{2}\|+\|{\ddot{\xi }}_{d}\|{}^{2}+\|{\dot{\xi }}_{d}\|{}^{2}\\ +\frac{1}{\beta^{2}}\|{\dot{p}}_{G}\| \end{array}\right)dt+\alpha^{2}\int_{0}^{T}\left(2p_{KR}+\epsilon_{0}\right)\|{\dot{\phi }}_{K}\|dt$$
  *as long as* ${\dot{\xi }}_{d}$ *is square integrable and* $\dot{\phi }$ *is absolutely integrable.* $\hat{p}$ *is the vector of estimates of the unknown parameters, which is updated by an adaptation law,*
*(iii)*  *when the disturbance is zero at all times,* $\dot{p}=0$ *and* ${\dot{\xi }}_{d}={\ddot{\xi }}_{d}=0$ *(the regulation control problem),* $\tilde{x}$ *converges to zero asymptotically.*

Note that if $\tilde{x}$ tends to zero, ξ and $\dot{\xi }$ converge to zero, too.

We introduce the control law as:

(36) $$U=M\left(p_{MN}\right){\ddot{\xi }}_{r}+G\left(p_{GN}\right)\dot{\xi }+K\left({\hat{p}}_{K}\right)\xi -R^{-1}{\tilde{x}}_{1}$$

As stated before, the unknown parameters enter linearly in the system’s equations, so two new matrixes $\Gamma_{G}$ and $\Gamma_{K}$ are defined as

(37) $$\Gamma_{K}\left(\xi \right){\hat{p}}_{K}=K\left({\hat{p}}_{K}\right)\xi$$

In the FGM micro-beam system $\Gamma_{K}$ can be obtained as

(38) $$\Gamma_{K}\left(\xi \right)=\left[K_{1},K_{2},K_{3}\right]$$

Also, the adaption law for the system is defined as

(39) $$\dot{\hat{p}}=A\;Proj\left(-\Gamma_{K}^{T}\left(\xi \right){\tilde{x}}_{2},{\hat{p}}_{K}\right)$$

where A>0 is an arbitrary matrix that satisfies the relation $\sigma \left(A^{-1}\right)=\alpha^{2}/2,$ and σ(.) is maximum singular value of a matrix.

$Proj\left(z,\hat{p}\right)$ is the smooth projection as

(40) $$Proj\left(z,\hat{p}\right)=\{\begin{array}{cc} z & if\;𝒫\left(\hat{p}\right)\leq 0\\ z & if\;𝒫\left(\hat{p}\right)\leq 0\;and\;{𝒫}^{\prime }\left(\hat{p}\right)z\leq 0\\ \left[I-\frac{𝒫\left(\hat{p}\right){𝒫}^{\prime }{\left(\hat{p}\right)}^{T}{𝒫}^{\prime }\left(\hat{p}\right)}{\|{𝒫}^{\prime }{\left(\hat{p}\right)\|}^{2}}\right]z & otherwise \end{array}$$

where

(41) $$𝒫\left(\hat{p}\right)=\frac{{\left(\hat{p}-p_{N}\right)}^{T}\left(\hat{p}-p_{N}\right)-p_{R}^{2}}{\epsilon^{2}+2\;\epsilon \;p_{N}}$$

and ${𝒫}^{\prime }\left(\hat{p}\right)=d𝒫\left(\hat{p}\right)/d\hat{p}$. I is the identity matrix of appropriate size and ϵ is an arbitrary positive real number. The value of ϵ affects the adaptation’s convergency speed. It should be mentioned that the adaptive law would not let the estimate $\hat{p}$ leave the compact set $\|p-p_{N}\|\leq p_{R}$.

**Theorem** **1.** Consider the system (30). By choosing the control law (36) alongside the adaptation law (39), then the adaptive control problem is stable as described in Definition 1.

**Proof.**  Consider the positive definite function

(42) $$V=\frac{1}{2}{\tilde{x}}_{2}^{T}M{\tilde{x}}_{2}+\frac{1}{2}{\tilde{x}}_{1}^{T}K{\tilde{x}}_{1}+\frac{1}{2}{\tilde{p}}_{K}A^{-1}{\tilde{p}}_{K}$$

where ${\tilde{p}}_{K}={\hat{p}}_{K}-p_{K}$. The derivative of the function V with respect to time is

(43) $$\begin{array}{cc} \dot{V}={\tilde{x}}_{2}^{T}M{\dot{\tilde{x}}}_{2}+ & {\tilde{x}}_{1}^{T}K{\dot{\tilde{x}}}_{1}+{\tilde{p}}_{K}^{T}A^{-1}\left({\dot{\hat{p}}}_{K}-{\dot{p}}_{K}\right)\\ & \;={\tilde{x}}_{2}^{T}M\left(p_{MN}\right){\ddot{\xi }}_{r}-{\tilde{x}}_{2}^{T}M\left(p_{M}\right){\ddot{\xi }}_{r}+{\tilde{x}}_{2}^{T}G\left(p_{GN}\right)\dot{\xi }-{\tilde{x}}_{2}^{T}G\left(p_{G}\right)\dot{\xi }+{\tilde{x}}_{2}^{T}\left(-R^{-1}{\tilde{x}}_{1}+\omega \right)\\ & \;+{\tilde{x}}_{1}^{T}K{\dot{\tilde{x}}}_{1}+{\tilde{x}}_{2}\Gamma_{K}{\tilde{p}}_{K}+{\tilde{p}}_{K}^{T}A^{-1}\left({\dot{\hat{p}}}_{K}-{\dot{p}}_{K}\right) \end{array}$$

If $\delta =\sigma \left(\left[\begin{array}{cc} \tilde{M} & \tilde{G} \end{array}\right]\right)$ then

(44) $${\tilde{x}}_{2}^{T}\tilde{M}{\ddot{\xi }}_{r}+{\tilde{x}}_{2}^{T}\tilde{G}\dot{\xi }={\tilde{x}}_{2}^{T}\left[\begin{array}{cc} \tilde{M} & \tilde{G} \end{array}\right]{\left[\begin{array}{cc} {\left(-\Lambda {\dot{\tilde{x}}}_{1}\right)}^{T} & {\dot{\tilde{x}}}_{1}^{T} \end{array}\right]}^{T}+{\tilde{x}}_{2}^{T}\tilde{M}{\ddot{\xi }}_{d}+{\tilde{x}}_{2}^{T}\tilde{G}{\dot{\xi }}_{d}\;\leq \frac{\delta^{2}}{2\epsilon_{1}^{2}}{\tilde{x}}_{2}^{T}{\tilde{x}}_{2}+\frac{\epsilon_{1}^{2}}{2}\left[\begin{array}{cc} {\dot{\tilde{x}}}_{1}^{T} & {\tilde{x}}_{1}^{T} \end{array}\right]\left[\begin{array}{cc} \Lambda^{T}\Lambda +I & 0\\ 0 & \Lambda^{T}\Lambda \end{array}\right]\left[\begin{array}{c} {\dot{\tilde{x}}}_{1}\\ {\tilde{x}}_{1} \end{array}\right]+\frac{\delta^{2}}{2\epsilon_{1}^{2}}{\tilde{x}}_{2}^{T}{\tilde{x}}_{2}+\frac{\epsilon_{1}^{2}}{2}\|{\ddot{\xi }}_{d}\|{}^{2}+\frac{\epsilon_{1}^{2}}{2}\|{\dot{\xi }}_{d}\|{}^{2}$$

where $\tilde{G}=G\left(p_{GN}\right)-G\left(p_{G}\right)$. Equation (39) has these properties [48]

(a) $\|\hat{p}-p_{N}\|\leq p_{R}+\epsilon \;for\;any\;t\geq 0$
(b) $Proj\left(z,\hat{p}\right)$ is Lipschitz and continuous
(c) $\|{\tilde{p}}^{T}Proj\left(z,\hat{p})\right\|\leq \tilde{p}z$
(d) ${\tilde{p}}^{T}Proj\left(z,\hat{p}\right)\leq \tilde{p}z$.

If the initial estimate ${\hat{p}}_{K}\left(0\right)$ is in the neighborhood $\|{\hat{p}}_{K}\left(0\right)-p_{KN}\|\leq p_{KM}$ and ${\dot{\hat{p}}}_{K}=A\;Proj\left(z,{\hat{p}}_{K}\right)$; then by virtue of property (d),

(45) $${\tilde{p}}_{K}^{T}\left(\Gamma_{K}{\tilde{x}}_{2}+A^{-1}{\dot{\hat{p}}}_{K}\right)\leq 0$$

Using Equations (44) and (45), we derive

(46) $$\dot{V}\leq \frac{\delta^{2}}{\epsilon_{1}^{2}}{\tilde{x}}_{2}^{T}{\tilde{x}}_{2}+\frac{\epsilon_{1}^{2}}{2}\left[\begin{array}{cc} {\dot{\tilde{x}}}_{1}^{T} & {\tilde{x}}_{1}^{T} \end{array}\right]\left[\begin{array}{cc} \Lambda^{T}\Lambda +I & 0\\ 0 & \Lambda^{T}\Lambda \end{array}\right]\left[\begin{array}{c} {\dot{\tilde{x}}}_{1}\\ {\tilde{x}}_{1} \end{array}\right]\;+\frac{\epsilon_{1}^{2}}{2}\left({\left\|\ddot{\xi }\right\|}_{d}{}^{2}+\|{\dot{\xi }}_{d}\|{}^{2}\right)+{\tilde{x}}_{2}^{T}\left(-R^{-1}{\tilde{x}}_{1}+\omega \right)+{\tilde{x}}_{1}^{T}K\tilde{x}-{\tilde{p}}_{K}^{T}A^{-1}{\dot{p}}_{K}$$

We introduce a new variable γ less than $\epsilon_{1}$ that satisfies Riccati-like inequality:

(47) $$\left[\begin{array}{cc} 0 & K\\ K & 0 \end{array}\right]+\epsilon_{1}^{2}\left[\begin{array}{cc} \Lambda^{T}\Lambda +I & 0\\ 0 & \Lambda^{T}\Lambda \end{array}\right]+Q-\left[\begin{array}{c} I_{n}\\ \Lambda^{T} \end{array}\right]\left(R^{-1}-\frac{2\delta^{2}+1}{\epsilon_{1}^{2}}I_{n}\right)\left[\begin{array}{cc} I_{n} & \Lambda \end{array}\right]\leq 0$$

where K>0 is an arbitrary matrix. This is a linear matrix inequality, which could be solved by iterative numerical methods.

Using Equation (47), it could be concluded that the new defined function V satisfies the following inequality

(48) $$\begin{array}{cc} V=\dot{V}+\frac{1}{2}({\tilde{x}}^{T}Q\tilde{x} & +{\left(R^{-1}{\tilde{x}}_{1}\right)}^{T}R\left(R^{-1}{\tilde{x}}_{1}\right))-\frac{1}{2}\alpha^{2}\;\left(2p_{KM}+\epsilon \right)\|{\dot{p}}_{K}\|\\ & -\frac{1}{2}\gamma^{2}\left({\left\|\omega \right\|}^{2}+\|{\ddot{\xi }}_{d}\|{}^{2}+\|{\dot{\xi }}_{d}\|{}^{2}+\frac{1}{\beta^{2}}\|{\dot{p}}_{G}\|{}^{2}\right)\\ & \leq \frac{\delta^{2}}{\epsilon_{1}^{2}}{\tilde{x}}_{2}^{T}{\tilde{x}}_{2}+\frac{\epsilon_{1}^{2}}{2}\left[\begin{array}{cc} {\dot{\tilde{x}}}_{1}^{T} & {\tilde{x}}_{1}^{T} \end{array}\right]\left[\begin{array}{cc} \Lambda^{T}\Lambda +I & 0\\ 0 & \Lambda^{T}\Lambda \end{array}\right]\left[\begin{array}{c} {\dot{\tilde{x}}}_{1}\\ {\tilde{x}}_{1} \end{array}\right]\\ & +\frac{1}{2}\left(\epsilon_{1}^{2}-\gamma^{2}\right)\left(\|{\ddot{\xi }}_{d}\|{}^{2}+\|{\dot{\xi }}_{d}\|{}^{2}\right)-{\tilde{x}}_{2}^{T}R^{-1}{\tilde{x}}_{1}+\frac{1}{2}{\left(R^{-1}{\tilde{x}}_{1}\right)}^{T}R\left(R^{-1}{\tilde{x}}_{1}\right)\\ & +{\tilde{x}}_{2}^{T}\omega -\frac{1}{2}\gamma^{2}{\left\|\omega \right\|}^{2}+\frac{1}{2}\left[\begin{array}{cc} {\dot{\tilde{x}}}_{1}^{T} & {\tilde{x}}_{1}^{T} \end{array}\right]\left(\left[\begin{array}{cc} 0 & K\\ K & 0 \end{array}\right]+Q\right)\left[\begin{array}{c} {\dot{\tilde{x}}}_{1}\\ {\tilde{x}}_{1} \end{array}\right]-{\tilde{p}}_{K}^{T}A^{-1}{\dot{p}}_{K}\\ & -\frac{1}{2}\alpha^{2}\;\left(2p_{KM}+\epsilon \right)\|{\dot{p}}_{K}\| \end{array}$$

Using the property (a) of the adaptation algorithm,

(49) $${\tilde{p}}_{K}^{T}A^{-1}{\dot{p}}_{K}-\frac{1}{2}\alpha^{2}\;\left(2p_{KM}+\epsilon \right)\|{\dot{p}}_{K}\|\leq \|{\tilde{p}}_{K}\|\sigma \left(A^{-1}\right)\|{\dot{p}}_{K}\|-\frac{1}{2}\alpha^{2}\;\left(2p_{KM}+\epsilon \right)\|{\dot{p}}_{K}\|\;\leq \sigma \left(A^{-1}\right)\left(2p_{KM}+\epsilon \right)\|{\dot{p}}_{K}\|-\frac{1}{2}\alpha^{2}\;\left(2p_{KM}+\epsilon \right)\|{\dot{p}}_{K}\|=\left(\sigma \left(A^{-1}\right)-\frac{1}{2}\alpha^{2}\right)\left(2p_{KM}+\epsilon \right)\|{\dot{p}}_{K}\|=0$$

because it was set that $\sigma \left(A^{-1}\right)=\frac{1}{2}\alpha^{2}$. On the other hand,

(50) $${\tilde{x}}_{2}^{T}\omega -\frac{\gamma^{2}}{2}\|\omega^{2}\|=-\frac{\gamma^{2}}{2}\|\omega -{\frac{{\tilde{x}}_{2}}{\gamma^{2}}\|}^{2}+\frac{1}{2\gamma^{2}}\|{\tilde{x}}_{2}\|{}^{2}$$

As a consequence of these recent results and substituting them in Equation (48),

(51) $$V\leq \frac{1}{2}x^{T}Qx\leq 0$$

The inequality (35) in Definition 1 is a result of integrating the above equation.

If $\omega ={\dot{\xi }}_{d}={\ddot{\xi }}_{d}=\dot{p}=0$, then

(52) $$\dot{V}\leq -\frac{1}{2}x^{T}Qx$$

As stated before, matrix M is bounded and positive definite, so that there exists a $K_{\infty }$ function $k_{l}\left(x,\phi \right)$ and $k_{u}\left(x,\phi \right)$ that satisfy [49]

(53) $$k_{l}\left(\|x\|,\|\phi \|\right)\leq V\left(\|x\|,\|\phi \|\right)\leq k_{u}\left(\|x\|,\|\phi \|\right)$$

The result is that x is bounded. The boundedness of ϕ is a result of the adaptive projection law. By using Barbalat’s lemma, it could be concluded that x converges to zero with time. ☐

**Remark** **1.** *It should be noted that by using this control algorithm, only the unknown parameters in the matrix* K *are being updated. So the normalized parameter* $\alpha_{4}$ *is not being updated, but, the parameters* $\alpha_{2}$, $\alpha_{3}$ *and* $\alpha_{5}$ *are being updated. The results of simulating the estimations updates will be presented in the next section.*

## 4. Simulations and Discussion

In this section, the results of numerous numerical simulations are presented to demonstrate the performance of the proposed active control algorithm for the functionally graded material micro-switch.

Table 2 lists the material properties used in the simulations, and Table 3 specifies the geometric parameters of the micro-switch. As noted before, the parameters of the system are unknown but the maximum errors compared with the actual values are known. As can be seen from Table 3, the dielectric gaps for the bottom and top electrodes are not the same.

### 4.1. Safe Region for Applied Voltage

The validity of the model presented by Equation (2) is verified by comparing the simulation results with the experimental data.

**Figure 2:**
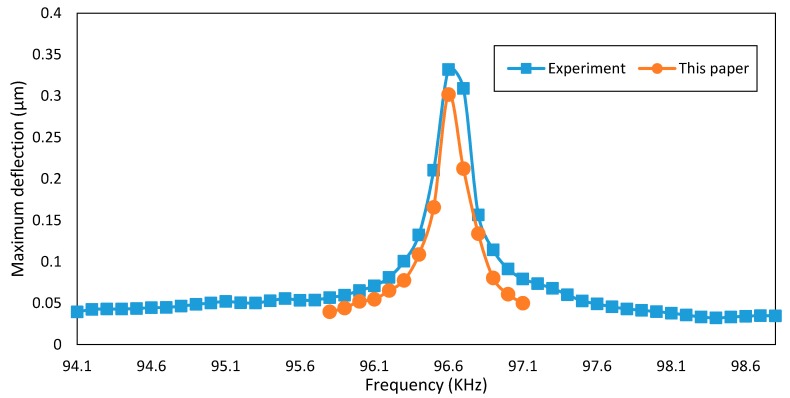
Comparison of the results of numerical simulations in this paper with the experimental results [50].

Ebrahim et al. [50] found the maximum amplitude of a clamped-clamped micro-beam of 600 μm length. The applied voltage is V=3+2 cos(ωt), in which ω is the oscillation frequency. The equivalent rigidity of the micro-beam is $2.93\times 10^{-11}N\cdot m^{2}$, the non-dimensional axial force is 76.3, the damping ratio is $6\times 10^{-4}$, and the initial gap between two electrodes is 2 μm. The results of numerical simulations (five modes approximation) of this paper are compared with the experimental results and are presented in Figure 2.

The response of the system when the applied voltage increases is also compared with experimental results demonstrated by Hu et al. [51]. Table 4 presents the tip deflection of a clamped-free micro-beam (five modes approximation) with L=20 mm, h=54 μm, E=155.8 Gpa and the initial gap is 92 μm.

As can be seen from Figure 2 and Table 4, the model presented in this paper is in good agreement with various experimental results.

### 4.2. Safe Region for Applied Voltage

The electrostatic actuation of micro-beams results in highly nonlinear dynamics, leading to a saddle-node bifurcation, called pull-in. The performance of electrostatic actuators is severely limited by the pull-in instability, which is due to electrostatic force increasing more rapidly than the spring force of the micro-beam [52]. In the active vibration control of micro-beams using electrostatic actuation, the value of pull-in voltage should be computed. Applied voltage of electrodes should be saturated to avoid the pull-in instability. For this reason, the controller gains are chosen such that the applied voltage of each electrode is less than 90% of the pull-in voltage. Table 5 presents the pull-in voltage for clamped-clamped and clamped-free boundary conditions for different volume fraction indices. It should be noted that the values in Table 5 are static pull-in voltages that are larger than pull-in voltages in a controlled system [53].

### 4.3. Multimode Simulation of the Controlled System in Regulation

**Figure 3:**
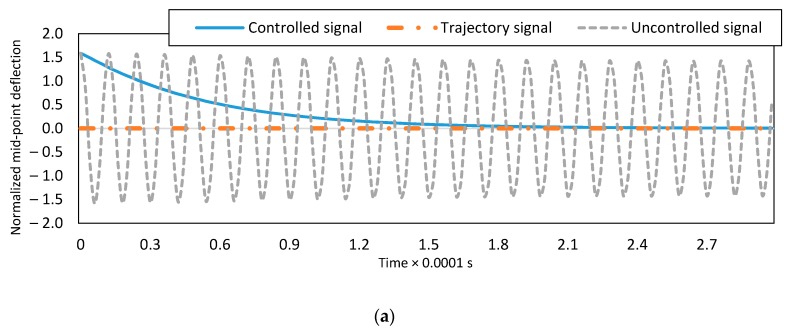

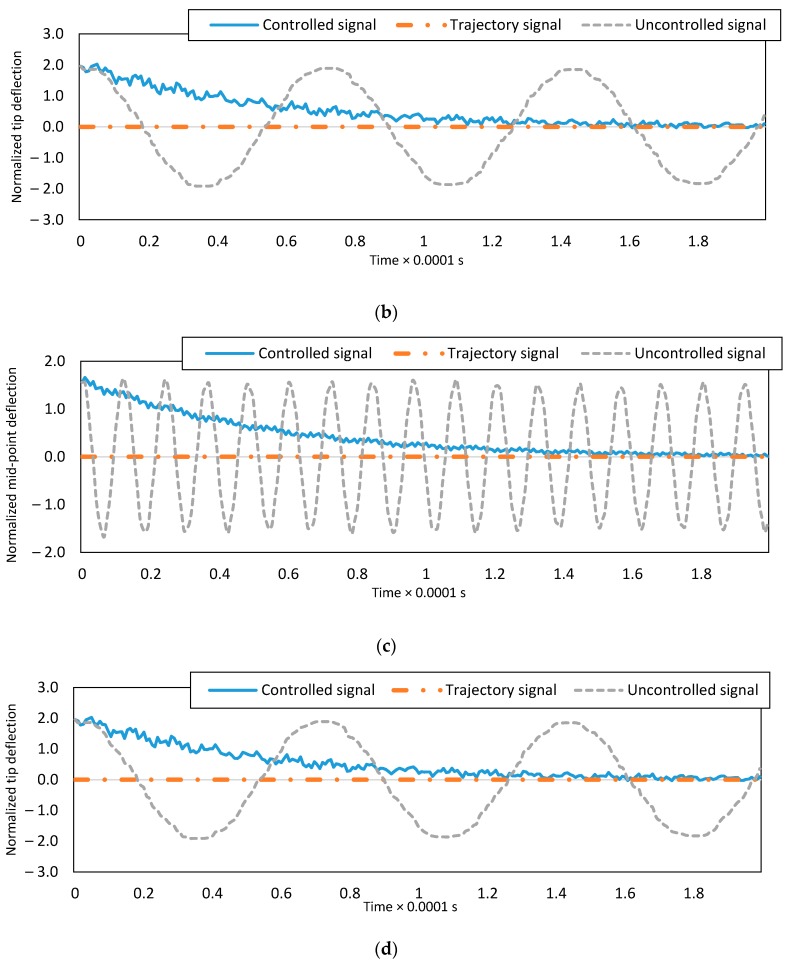
Controlled and uncontrolled dynamic response using one mode approximation for: (**a**) clamped-clamped and (**b**) clamped-free boundary conditions and five modes approximation for: (**c**) clamped-clamped and (**d**) clamped-free boundary conditions.

**Figure 4:**
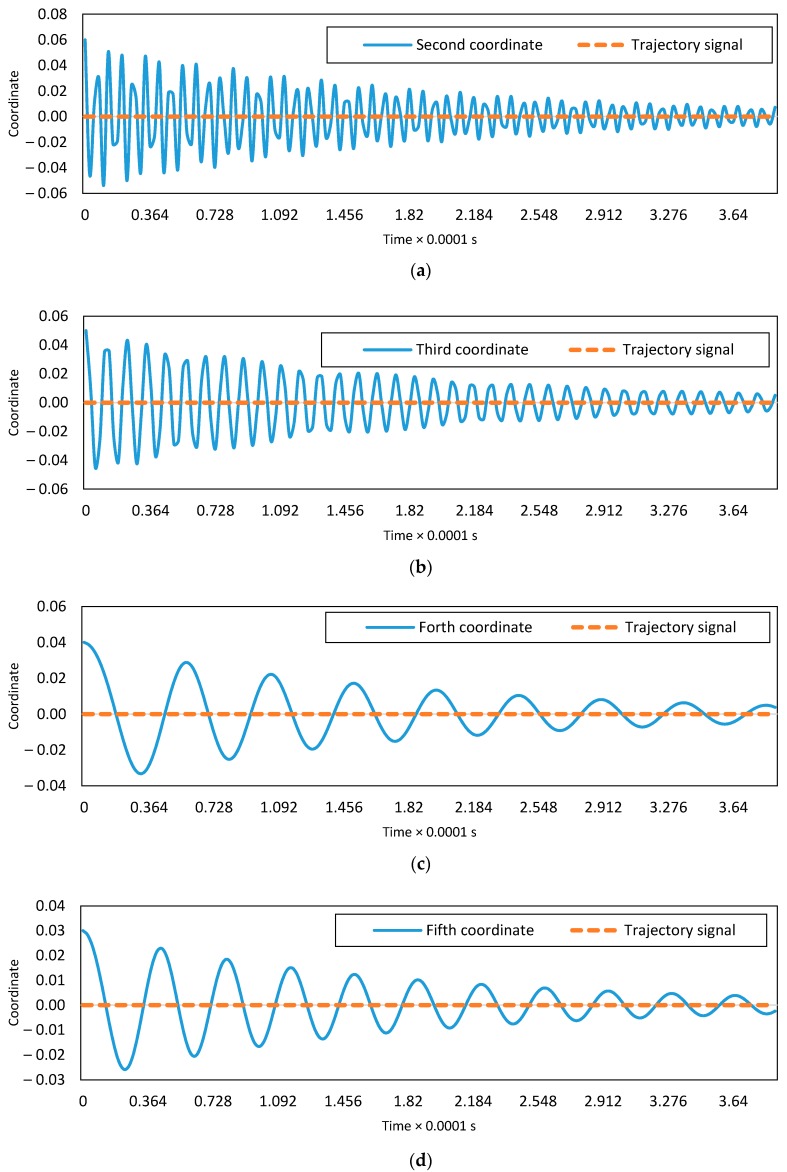
Desired path and controlled time response of second to fifth modes for clamped-clamped boundary condition: (**a**) the second mode, (**b**) the third mode, (**c**) the fourth mode and (**d**) the fifth mode.

The uncontrolled dynamic and controlled responses of the system for the mid-point of C-C and end-point of C-F boundary conditions are presented in Figure 3. The controlled system response converges to the desired trajectory $\xi_{d}=0$ in less than $3\times 10^{-4}$ s. The design of the presented controller is based on the first two vibrational modes. To show the proper performance of the designed controller, higher vibration modes of the system are considered and the five-mode approximation responses of the uncontrolled and controlled systems are demonstrated in Figure 3c,d. The initial estimates of dimensionless parameters have 5% error. It is seen that the nonlinear vibrations are suppressed via the imposed controller. The dynamic response of the second to fifth coordinates for the C-C boundary conditions are shown in Figure 4. All the coordinates converge to zero, which is the desired signal.

**Figure 5:**
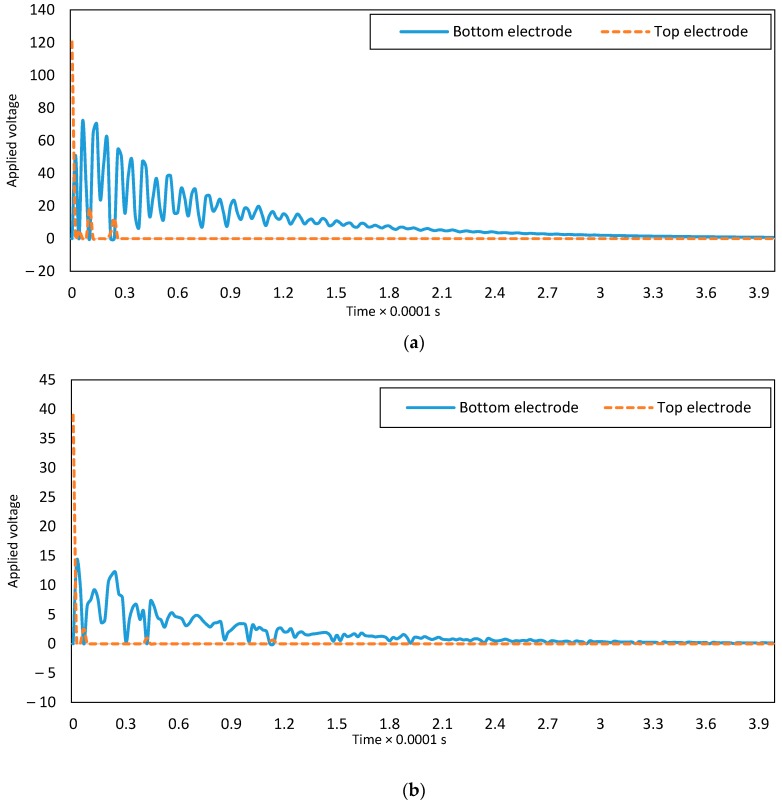
Supplied voltage of two electrodes for: (**a**) clamped-clamped and (**b**) clamped-free boundary conditions.

Figure 5 depicts the voltage values of the two electrodes. Based on the proposed control algorithm, only one of the voltage values is non-zero to decrease the control efforts.

**Figure 6:**
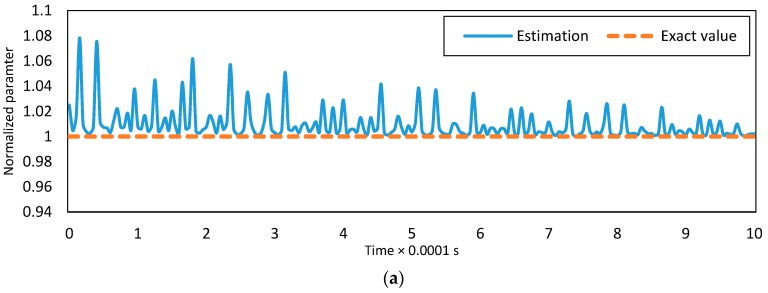

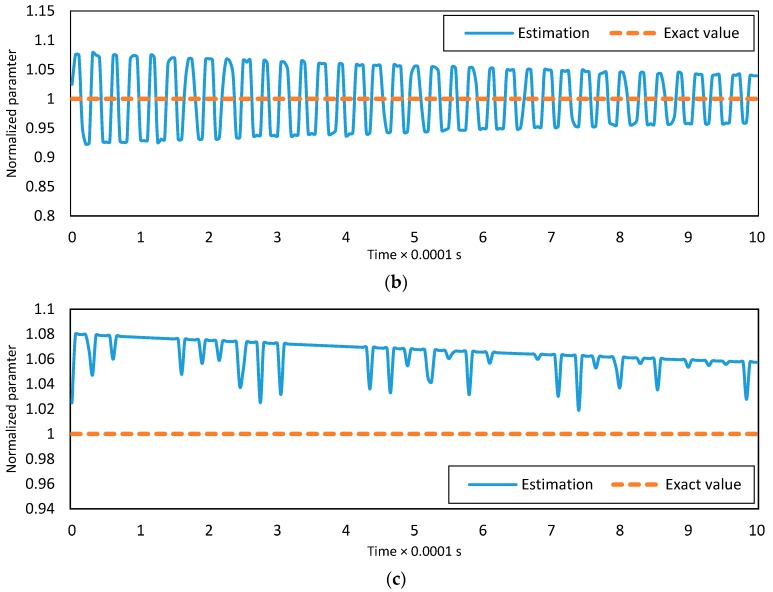
Estimation of the parameters; (**a**) $\alpha_{1}$, (**b**) $\alpha_{2}$, and (**c**) $\alpha_{3}$.

The adaptive projection law is updating estimates of the unknown dimensionless parameters. The estimates belong to the known closed sets at all times. If the desired signal has enough dissimilar frequencies, the estimates will converge to exact values [54]. The necessary number of frequencies can be found by analyzing the complexity of the system. In the case of this paper, the desired signal should have seven or more dissimilar frequencies such that the estimates of parameters converge to exact values. This is a result of studying the results of various simulations. It should also be mentioned that the measure of unmodeled forces would affect the convergence rate. The estimations of $\alpha_{1}$, $\alpha_{2}$ and $\alpha_{4}$ are plotted for the C-F boundary conditions in Figure 6 to show how the adaptive projection law operates.

### 4.4. Tracking the Non-Zero Desired Dynamical Response

**Figure 7:**
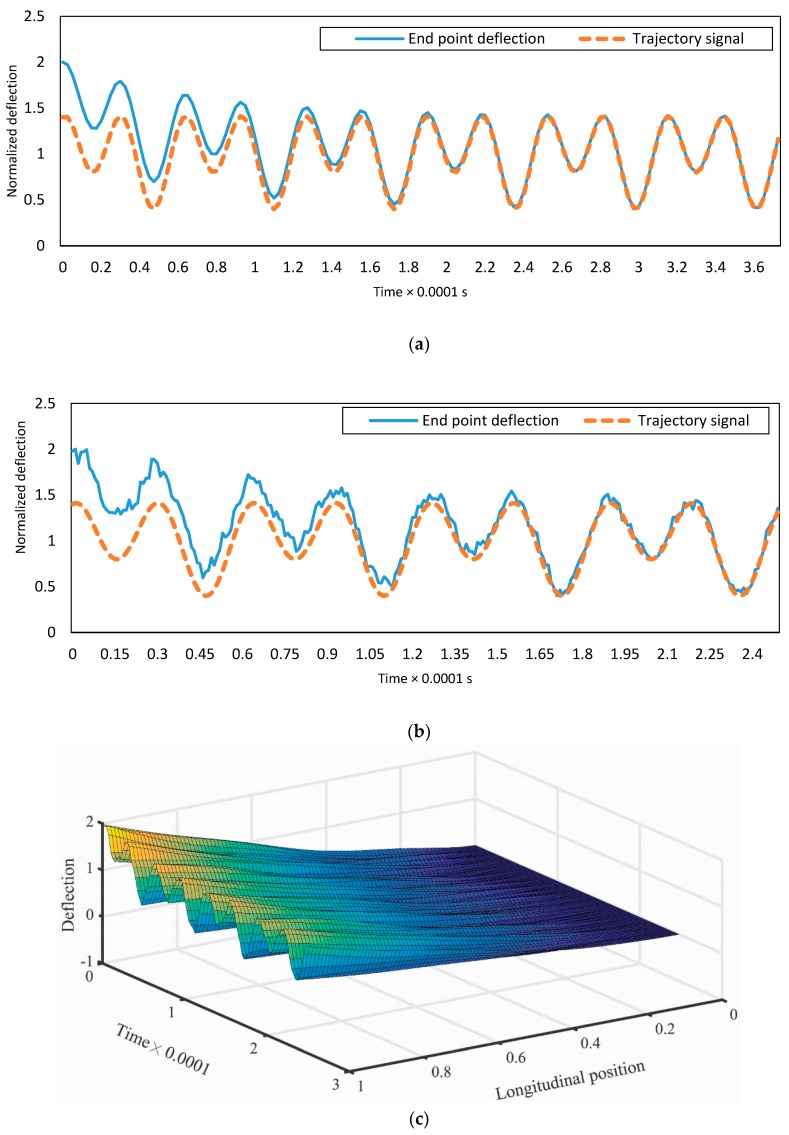
(**a**) Tip deflection of the clamped-free micro-switch for using the first mode; (**b**) tip deflection of the clamped-free micro-switch for using the first five modes; and (**c**) the deflection of the clamped-free micro-switch.

Figure 7 depicts the dynamic responses of the end-point deflection of the C-F micro-switch for one mode and five modes approximation of the controlled system. The desired signal is considered as

(54) ξ(t)=0.5+0.1sin(10 t)+0.2cos(20 t)

Two vibrational modes are used to obtain the control law. In this study, n=1. The error of the initial estimates of the parameters is 10%. The disturbance is a periodic signal whose amplitude is a random multiplier from 0.5 to 0.8 of the designed input and its period is three times smaller than the first natural frequency of the C-F micro-switch. The displacement of all points of the micro-switch is simulated by five modes and presented in Figure 7c. In this three-dimensional figure, the switch displacement is plotted as a function of axial position and time of the micro-switch. This figure depicts how the micro-switch is deflected through time.

### 4.5. Energy Consumption

**Figure 8:**
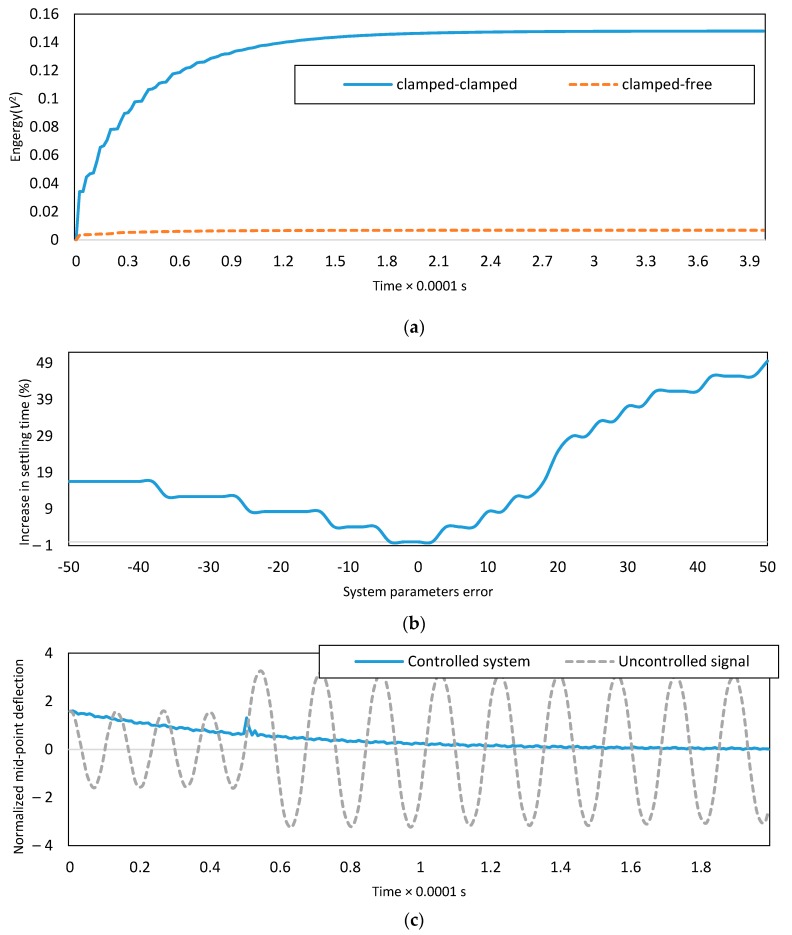

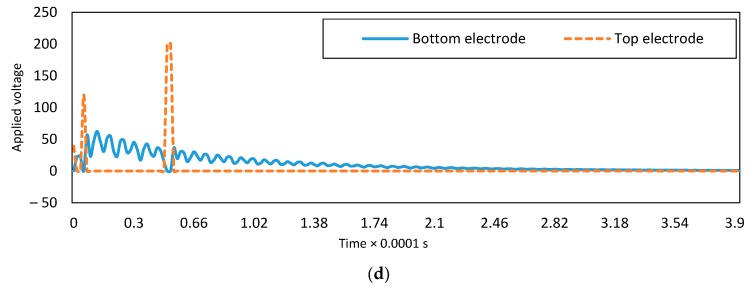
(**a**) Applied energy for tracking zero desired path; (**b**) increase in the settling time due to uncertainties in the system parameters; (**c**) the dynamic response of the clamped-clamped micro-switch subjected to an impulse; and (**d**) supply voltage of two electrodes under the effect of an impulse.

The applied energy for two micro-switches with different boundary conditions is plotted in Figure 8a. The energy is defined as $\int V^{2}dt$. It can be seen that the consumption of energy is less for clamped-clamped switch, which is due to the higher structural stiffness of this micro-switch. 

### 4.6. Effect of Error of Initial Estimates of the Parameters

If the errors in initial estimates of the dimensionless parameters increase, the 5% settling time (when the dynamic response of the system is in the 5% neighborhood of the desired trajectory) of the system response will also increase. The effect of the error of initial estimates on the increasing settling time is investigated in Figure 8b. If the error of initial estimates of dimensionless parameters is 50%, the settling time of the system will be 50% higher. The adaptive projection law would compensate for the error of initial estimates of the parameters as long as the bounds for these parameters are known. However, the estimates may not converge to the exact values. 

As a design tip, as the error increases, the adaptation gains should be lowered; otherwise, a higher frequency measurement device is needed. The low-frequency measurement device, high adaptation gains, and significant errors lead to an unstable controlled system.

### 4.7. Effect of Impulse on the System

The effect of the impulse is investigated in Figure 8c,d. The dynamic response of the uncontrolled system and the controlled system for C-C boundary conditions is shown in Figure 8c. The value of impulse is $6.92\times 10^{-3}\;N\cdot s$, which is applied at t=0.5. The higher values of impact lead to unacceptable deflections. The proposed active vibration control algorithm could absorb the vibration due to the impulse and initial conditions. Figure 8c depicts the dynamic response of the controlled system when the impulse value is $6.92\times 10^{-2}N\cdot s$, and the desired signal is equal to zero. The boundary conditions are C-C, and the error of the initial estimates of the dimensionless parameters is 10%. The supplied voltages of the two electrodes for the controlled system are demonstrated in Figure 8d. As shown in Figure 8d, the controlled system could compensate for the effect of impulse by regulating the applied voltages of the electrodes instantly.

### 4.8. Effect of Material Volume Fraction

**Figure 9:**
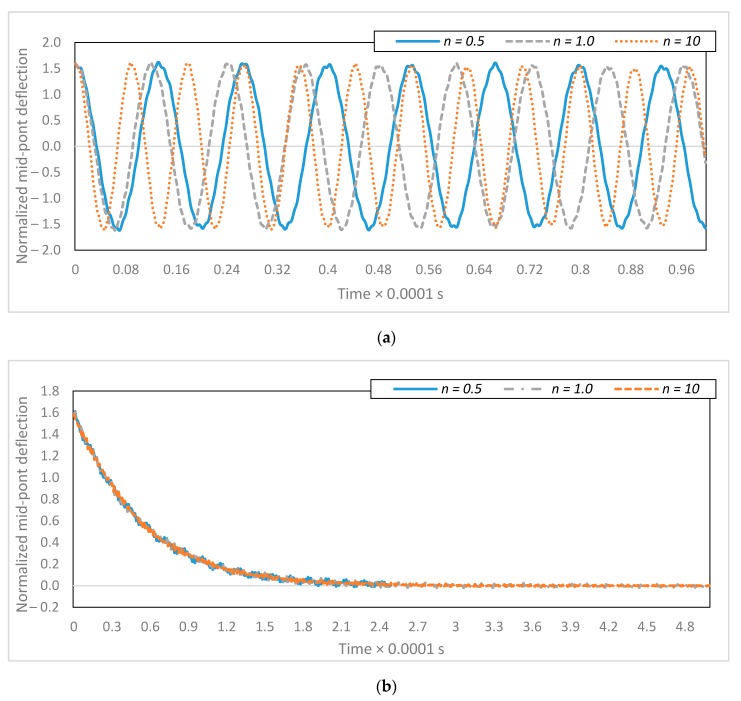
Deflection of the clamped-clamped micro-switch for various volume fractions: (**a**) the uncontrolled system and (**b**) the controlled system.

As the volume fraction index increases, the stiffness of the material increases, too. Figure 9a demonstrates the dynamic response of the uncontrolled C-C micro-switch for different volume fractions. The vibration suppression of the system is plotted in Figure 9b for three various volume fraction indices. In the case of this study, the desired signal is zero. As can be seen from Figure 9b, the dynamic response of the uncontrolled system depends on the volume fraction index, while the dynamic response of the controlled system is the same for various volume fraction indices. The active vibration control strategy is robust to changes in material properties as long as the initial estimates of the dimensionless parameters are not too inaccurate. The controlled system compensates for the change in material properties by adjusting the applied voltage of the two electrodes.

## 5. Conclusions

This paper has investigated the multi-mode robust active vibration control of the FG micro-switch. The modified couple stress theory, which introduces a length-scale parameter, was used to derive the governing equations of motion. The FGM micro-switch was under the influence of electrostatic and intermolecular forces. Two sets of boundary conditions for the micro-switch were considered: clamped-clamped and clamped-free. The discretized model of the partial differential equation of motion was obtained using the modal analysis method. An active vibration control algorithm was proposed to overcome the system parameter uncertainties and unmodeled forces, while the deflection of the micro-switch should converge to the desired dynamical response. The inputs of the controlled system were the voltage values of the two electrodes and a nonlinear robust adaptive controller based on the first two modes of vibrations was introduced. The simulations for higher modes were demonstrated to show the decent performance of the designed controller. For increasing usability of the presented micro-switch, many remarks were established. By introducing a performance signal, high gain input voltages were avoided. Also, the controller gains were chosen such that the static pull-in voltage was avoided, too.

The controller was designed for two modes (of the modal analysis method) approximation, while a five-mode approximation of deflection was used to simulate the dynamic response of the system to demonstrate the performance of the proposed active control algorithm. The controlled system can track non-zero dynamical behavior while the undesired vibrations were being suppressed.

The effects of the error of estimates of the system parameters were also studied. It was concluded that this increase would result in an increase in the settling time of the controlled system. In this case, the parameter estimation should be updated with a lower rate.

Simulations of dynamical responses of the system showed that larger volume fraction indices of FG materials led to higher natural frequencies. However, the controlled system was robust, and the dynamic response of the controlled system was almost the same for various volume fraction indices.

In addition, the vibrational response of the system after an impulse was studied. By comparing the dynamic response of the uncontrolled and controlled systems, it was concluded that the controlled system was more robust to impulses. The controlled system could compensate for the effect of the impulse by instantly regulating the applied voltages of the electrodes.

The phase portrait of each coordinate was plotted to demonstrate the stability of the controlled micro-switch. Each coordinate and its time derivative converged straight to zero except for the first modal coordinate, which followed the desired dynamical response.

## Figures and Tables

**Table 1:** The first five modes for clamped-clamped and clamped-free boundary conditions.

Boundary Conditions	$s_{1}$	$s_{2}$	$s_{3}$	$s_{4}$	$s_{5}$
C-C	4.7300	7.8532	10.9956	14.1371	17.2787
C-F	1.8751	4.6941	7.8548	10.9955	14.1372

**Table 2:** Material properties of the FG micro-switch.

Material Property	Silicon Nitride (Si_3_N_4_)	Nickel (Ni)
Density ($\mathrm{kg}/m^{3}$)	3200	8890
Young’s modulus (Pa)	$322.27\times 10^{9}$	$205.10\times 10^{9}$
Shear modulus (Pa)	$129.95\times 10^{9}$	$78.28\times 10^{9}$
Poisson’s ratio	0.24	0.31

**Table 3:** Geometric parameters of the FG micro-switch.

L	b	h	$l_{t}$	$l_{b}$	l
400 μm	10 μm	1 μm	8 μm	6 μm	0.2 μm

**Table 4:** Comparison of the results of numerical simulations in this paper with the experimental results [51].

Applied Voltage	This Paper	Experimental Data	Error (%)
0	92	92.415	0.451087
20	90.41261	91.018	0.669583
40	85.21468	84.232	1.153177
45	81.97923	81.437	0.661419
50	79.51302	78.643	1.09419
55	75.30798	74.651	0.872396
60	71.47106	70.06	1.974304
64	64.40234	62.874	2.373112
67	59.20658	57.485	2.907757
69	52.79904	51	3.407331

**Table 5:** Static pull-in voltage for C-C and C-F boundary conditions and various volume fractions.

Boundary Conditions	The Top Electrode			The Bottom Electrode		
	n=0.5	n=1	n=10	n=0.5	n=1	n=10
C-C	261.0 V	267.8 V	285.5 V	160.6 V	165.5 V	173 V
C-F	43.0 V	44.1 V	49.9 V	25.3 V	24.7 V	26.5 V
